# Supplementary material for: Identification of key regulators in pancreatic ductal adenocarcinoma using network theoretical approach
Source: PLoS One. 2025 Jan 27;20(1):e0313738. doi: 10.1371/journal.pone.0313738 (PMC11771905; doi:10.1371/journal.pone.0313738)
Supplement: Attachment — Submitted filename: Response to Reviewer.docx [file pone.0313738.s001.docx]

Dear Editor,

We would like to thank you and the reviewers for your insightful comments and positive reviews. We agree with the changes suggested by reviewers and have revised the manuscript as per their suggestions. I have also included our detailed response to the reviewers’ comments at the bottom of this letter.

**Kindly update financial disclosure statement** as “Indian Council of Medical Research (ICMR) (BMI/11(92)/2022).The funders had no role in study design, data collection and analysis, decision to publish, or preparation of the manuscript.”

Yours sincerely,

Aryya

Dr. Aryya Ghosh

Assistant Professor

Department of Chemistry

Ashoka University, 131029

**Reviewer #1:**

1. *Please use the abbreviations consequently. For example, PDAC is not always used in the Introduction. Introduce it at the first appearance and then use only the abbreviation.*

**We have made the necessary changes in the manuscript to indicate this fact.**

2. *Analyzing only 15 up-regulated DEGs for GO and KEGG in Figure 3 may not offer a complete understanding of the functional features of the entire genome, restricting the comprehensive insight into biological processes and cellular components. The limited sample size might lead to inadequate statistical significance of the findings, affecting the precise interpretation and inference of gene functions. It is advisable to perform GO and KEGG analysis using a larger set of DEGs, as shown in Figure 2. Furthermore, the text size and layout within Figure 2 and 3 should be uniform to maintain consistency in the analyses.*

**We agree with the reviewer's comment and clarified this fact in the text.**

**Fig. 3. Gene Ontology analysis for up-regulated genes of pancreatic ductal adenocarcinoma**

3. *The captions in Figure 4 are too brief; it is recommended to provide a brief description of the general meaning represented by each image. Additionally, the styles of A-F in Figure 4 are inconsistent with the other pictures, so it is suggested to modify them to a consistent format.*

**Thanks for comment. Necessary changes are made in manuscript as:**

**Fig 4: Topological properties of Network with power law fit. (A)** Probability of node degree distribution. **(B)** Clustering coefficient vs degree distribution. **(C)** Betweenness centrality vs degree distribution. **(D)** Closeness centrality vs degree distribution **(E)** Neighbourhood connectivity vs degree distribution. **(F)** Eigenvector value vs degree distribution.

4. *The simplicity of the description of Table 3-5 in the text belies the wealth of key information found through DGIdb, TargetScan, and miRTarBase databases. The significance of uncovering such a plethora of data is profound and warrants a detailed discussion in the main text.*

**We thank the reviewer for this comment. We have made the necessary changes in the manuscript to indicate this fact.**

Using DGIdb, we conducted a query to identify interactions between a predefined list of genes (33 key regulator genes) implicated in pancreatic ductal adenocarcinoma and known drugs. The key regulator gene list was derived from this study, and DGIdb was queried with approved drugs parameters, capturing interaction types such as inhibition, activation, and binding. We identified 91 drug-gene interactions involving 14 unique genes and 79 approved distinct drugs. Among these, genes MMP1, F10, F2R, GNRH1 had the highest number of interactions, interacting with more than 20 different drugs. The interactions included various types such as inhibition (50%), activation (30%), and binding (20%). The identification of MMP1, F10, F2R, GNRH1 as a highly interactive gene underscores its potential role in pancreatic ductal adenocarcinoma. These findings warrant further investigation to validate the interactions and explore their clinical applications.

We identified 15 key transcription factors, including NF1(neurofibromin 1), NFKB1(nuclear factor of kappa light polypeptide gene enhancer in B-cells 1), and STAT1(signal transducer and activator of transcription 3 (acute-phase response factor)), that regulate our genes of interest. Notably, NFKB1 was found to regulate a network of genes involved in positive regulation of protein kinase B signaling process or Cell adhesion molecules pathway, highlighting its potential role in pancreatic ductal adenocarcinoma.

MicroRNAs (miRNAs) are crucial post-transcriptional regulators that can influence gene expression by targeting transcription factors (TFs). In this study, we utilized the TARGET SCAN database to predict miRNAs that regulate TFs involved in pancreatic ductal adenocarcinoma. We identified several key miRNAs predicted to target transcription factors, including hsa-miR-1-3p, hsa-miR-335-5p, hsa-miR-526b-3p, hsa-miR-106b-5p, hsa-miR-17-5p and hsa-miR-124-3p. Notably, hsa-miR-335-5p was predicted to target 9 key genes with a high context score (**FDR=**0.619**, p-value**=0.043), suggesting a strong regulatory interaction. Additionally, hsa-miR-1-3p was predicted to target *RNF213, F2RL1, AP1S3*, indicating its potential role in regulating multiple pathways. The identification of hsa-miR-17-5p as a regulator of *F2RL1, F2R, TJP1* suggests a significant role in positive regulation of cell proliferation. The high context score (**FDR=**0.641**, p-value**=0.378) indicates a strong interaction, which could be critical for modulating positive regulation of cell proliferation.

*5. When addressing the functions of GO in the Discussion, it is crucial to start with MF, then CC, and finally BP, maintaining the sequence as shown in Table 6.*

**We thank the reviewer for this comment. We have made the necessary changes in manuscript.** *6. In the Discussion section, the author diligently discusses the roles of various key genes in previous studies, but most of them focus on other tumors. There is insufficient discussion on the molecular mechanisms related to PDAC. It is recommended to add relevant references and make modifications (doi: 10.3389/fgene.2023.1115660).*

**We thank the reviewer for this comment. We have made the necessary changes in the manuscript to indicate this fact as “**Previous research has shown that in PDAC cancer cells, the PAR2 protein is essential for TGF-β1-dependent cell motility (ref. 84). Cadherin-1 and cadherin-3 cooperation determines the aggressiveness of PDAC (ref. 85). Of 12 cases of  PDAC, 11 (92%) had positive immunostaining for claudin-4, and seven (58%) for claudin-1(ref. 86). To investigate the role of MMP-1 in human PDAC, T. Ito.et. al (ref. 87) conducted immunohistochemical analyses on tissue samples from 2 fetal pancreases, 5 normal pancreases, 6 cases of chronic pancreatitis, and 46 PDAC cases. Additionally, this examined the involvement of neuromedin U (NmU) in the pathogenesis of PDAC. Findings provide insights into the molecular mechanisms underlying PDAC development and progression, highlighting potential therapeutic targets for this malignancy (ref. 88)**.** PDAC is characterized by high expression of transforming growth factor (TGF)-β and the G protein-coupled receptor proteinase-activated receptor 2 (PAR2), the latter of which functions as a cell-surface sensor for serine proteinases associated with the tumour microenvironment(84).

Here is the list of added relevant references in the revised manuscript (84-88)

84.	Zeeh F, Witte D, Gädeken T, Rauch BH, Grage-Griebenow E, Leinung N, et al. Proteinase-activated receptor 2 promotes TGF-β-dependent cell motility in pancreatic cancer cells by sustaining expression of the TGF-β type I receptor ALK5. Oncotarget. 2016 Jul 5;7(27):41095–109.

85.	Siret C, Dobric A, Martirosyan A, Terciolo C, Germain S, Bonier R, et al. Cadherin-1 and cadherin-3 cooperation determines the aggressiveness of pancreatic ductal adenocarcinoma. Br J Cancer. 2018 Feb 20;118(4):546–57.

86.	Tsukahara M, Nagai H, Kamiakito T, Kawata H, Takayashiki N, Saito K, et al. Distinct expression patterns of claudin-1 and claudin-4 in intraductal papillary-mucinous tumors of the pancreas. Pathol Int. 2005 Feb;55(2):63–9.

87.	Ito T, Ito M, Shiozawa J, Naito S, Kanematsu T, Sekine I. Expression of the MMP-1 in human pancreatic carcinoma: relationship with prognostic factor. Mod Pathol. 1999 Jul;12(7):669–74.

88.	Rhim AD, Stanger BZ. Molecular biology of pancreatic ductal adenocarcinoma progression: aberrant activation of developmental pathways. Prog Mol Biol Transl Sci. 2010;97:41–78.

**Reviewer #2:** *The study “Identification of Key Regulators in Pancreatic Ductal Adenocarcinoma using Network Theoretical Approach" presents extensive work. Experiments, statistics, and other analyses are performed to a high technical standard and are described in sufficient detail. Here are the following comments:*

Table 3: Basis of Drug Selection and Bond Strength Description

*Please mention the rationale behind choosing the specific drugs listed in Table 3. It would be helpful to provide information on the criteria used for their selection, such as their relevance to pancreatic ductal adenocarcinoma, prior research findings, or therapeutic-potential. Additionally, specify the sources from which these drugs were selected. This could include databases, literature references, or clinical trial data.*

Using DGIdb, we conducted a query to identify interactions between a predefined list of genes (33 key regulator genes) implicated in pancreatic ductal adenocarcinoma and known drugs. The key regulator gene list was derived from this study, and DGIdb was queried with approved drugs parameters, capturing interaction types such as inhibition, activation, and binding. Drug-gene interactions are crucial in understanding disease mechanisms and identifying potential therapeutic targets. To explore these interactions in the context of pancreatic ductal adenocarcinoma, we utilized the Drug-Gene Interaction Database (DGIdb), which compiles data from multiple curated sources, including DrugBank and PharmGKB.

*It would also be beneficial to elaborate on the factors used to describe the strength of the bonds formed between the drugs and their targets. This could include binding affinity, interaction energy, or other relevant metrics. Providing this information will enhance the understanding of the drug-target interactions and their potential efficacy.*

Thanks for your insightful comments regarding binding affinity, interaction energy etc. This will be the future direction of our work.

*Manuscript Review: Typos and Spelling Mistakes
Kindly review the entire manuscript for any typographical and spelling errors. Ensuring that the text is free of such errors will improve the clarity.*

**We thank the reviewer for his helpful suggestion. We have gone through the manuscript and made the required changes.**
