# Supplementary material for: Identification of key regulators in pancreatic ductal adenocarcinoma using network theoretical approach
Source: PLoS One. 2025 Jan 27;20(1):e0313738. doi: 10.1371/journal.pone.0313738 (PMC11771905; doi:10.1371/journal.pone.0313738)
Supplement: Attachment — Submitted filename: Response to Reviewer-v2.docx [file pone.0313738.s002.docx]

Dear Editor,

We would like to thank you and the reviewers for your insightful comments and positive reviews. We agree with the changes suggested by reviewers and have revised the manuscript as per their suggestions. I have also included our detailed response to the reviewers’ comments at the bottom of this letter.

**Kindly update financial disclosure statement** as “Indian Council of Medical Research (ICMR) (BMI/11(92)/2022).The funders had no role in study design, data collection and analysis, decision to publish, or preparation of the manuscript.”

Yours sincerely,

Dr. Aryya Ghosh

Ashoka University, Department of Chemistry, Sonipat, Haryana -131029

E-mail: aryya.ghosh@ashoka.edu.in

**Reviewer #1:**

*6. In the Discussion section, the author diligently discusses the roles of various key genes in previous studies, but most of them focus on other tumors. There is insufficient discussion on the molecular mechanisms related to PDAC. It is recommended to add relevant references and make modifications (doi: 10.3389/fgene.2023.1115660).*

*Additional Editor Comments:*

*Please ensure that all comments from Reviewer #1 are thoroughly addressed. Specifically, the discussion on the molecular mechanisms associated with PDAC is lacking. Although the authors highlight pathways such as neuroactive ligand-receptor interaction and leukocyte transendothelial migration, it would be helpful to elucidate their relevance to PDAC development and progression. Can the authors provide a clearer connection between these pathways and the disease phenotype?*

**We thank the reviewer for this comment. We have made the necessary changes in the manuscript to indicate this fact as:**

The study showing the role of proteinase-activated receptor 2 (PAR2) in TGF-β1-dependent cell motility underlines the importance of PAR2 in the tumor microenvironment. The proteinase-activated receptor 2 (PAR2) is crucial for TGF-β1-dependent cell motility, establishing PAR2 as a key player in PDAC invasion and metastasis (84). PAR2 is a G protein-coupled receptor (GPCR) that senses extracellular proteases, particularly serine proteinases, which are abundant in the PDAC microenvironment. The interaction between PAR2 and TGF-β1 promotes tumor cell movement, a process essential for invasion into surrounding tissues and metastasis, which are hallmarks of PDAC. High expression of TGF-β1 further contributes to immune evasion and the development of a dense stromal environment, which creates barriers to effective therapy. TGF-β1 is well-known for its dual role in cancer, functioning as a tumor suppressor in early-stage cancers and as a promoter of invasion and metastasis in later stages, particularly in PDAC. The cooperation between cadherin-1 (E-cadherin) and cadherin-3 (P-cadherin), both critical cell adhesion molecules, plays a pivotal role in determining PDAC aggressiveness (85). While E-cadherin downregulation promotes epithelial-mesenchymal transition (EMT), a key process in cancer metastasis, P-cadherin overexpression in PDAC may exacerbate this effect, leading to increased tumor invasiveness. This interplay highlights the molecular dysregulation of adhesion mechanisms in PDAC, facilitating the cancer's spread to distant sites. The research showing that 92% of PDAC cases had positive immunostaining for claudin-4 and 58% for claudin-1 (86) suggests that claudins are critical markers in PDAC pathology. Claudins are integral components of tight junctions, which regulate paracellular permeability and maintain epithelial barrier function. In PDAC, the overexpression of claudins, particularly claudin-4, has been associated with tumor invasion and metastasis. These finding positions claudin-4 as a potential biomarker for PDAC, with therapeutic implications given its overexpression in a majority of PDAC tumors. Claudin-4 may also influence the permeability of the tumor microenvironment, facilitating cancer cell dissemination. The study by T. Ito et al. (87) focused on matrix metalloproteinase-1 (MMP-1), which plays a crucial role in tissue remodeling and degradation of the extracellular matrix (ECM). MMP-1 is frequently upregulated in cancer and contributes to tumor invasion and metastasis by breaking down the ECM barriers that normally confine cells. In the analysis of PDAC tissues, MMP-1 was found to be significantly elevated, suggesting its involvement in PDAC progression, particularly in enabling tumor cells to invade surrounding tissues. This highlights MMP-1 as a potential therapeutic target, where inhibiting its activity might reduce the invasive capabilities of PDAC cells. Additionally, neuromedin U (NmU) has been implicated in PDAC pathogenesis, providing novel insights into its role in cancer biology (88). NmU, a neuropeptide known for its role in immune response and regulation of smooth muscle contraction, appears to promote tumor growth and metastasis in PDAC. The upregulation of NmU in PDAC tissues correlates with more aggressive tumor behavior, suggesting that NmU-targeted therapies could offer new avenues for treating PDAC, particularly in cases where NmU expression is high. Another significant finding in PDAC research is the role of microRNA miR-7-5p, which acts as a tumor suppressor by targeting SOX18, a transcription factor involved in angiogenesis and EMT (89). The downregulation of miR-7-5p in PDAC leads to SOX18 overexpression, promoting tumor progression through enhanced angiogenesis and increased metastatic potential. Restoring miR-7-5p levels could inhibit tumor growth and reduce metastasis, offering potential therapeutic strategies focused on microRNA-based therapies in PDAC.

The neuroactive ligand-receptor interaction pathway primarily involves neurotransmitters and their receptors, which traditionally mediate signaling in the nervous system. However, increasing evidence indicates that these molecules also have significant roles in tumor biology, including PDAC. Several neurotransmitters, such as serotonin, dopamine, and norepinephrine, are known to interact with their respective receptors on pancreatic cancer cells, influencing signaling pathways that promote tumor cell survival, proliferation, and metastasis. For example: Serotonin receptors (5-HT receptors) have been found to be overexpressed in PDAC, enhancing tumor growth. Serotonin signaling activates downstream oncogenic pathways such as the PI3K/AKT and RAS/ERK pathways, which promote cell proliferation and survival. Inhibition of these receptors or signaling pathways has shown to reduce PDAC cell proliferation, indicating a direct role of neurotransmitter-receptor interactions in the tumor’s growth dynamics (90). One of the hallmark features of PDAC is perineural invasion (PNI), where cancer cells spread along nerve fibers. Neuroactive ligand-receptor interactions are central to this process, as tumor cells communicate with nerve cells to enhance their migration along these neural pathways. Perineural invasion in PDAC correlates with worse prognosis and increased metastasis, especially since neural invasion facilitates access to distant organs (91). Cancer cells exploit neurotransmitter signaling to promote their affinity for nerves. This invasion is mediated by neurotrophic factors, such as nerve growth factor (NGF) and glial cell-derived neurotrophic factor (GDNF), which bind to their receptors (TrkA and RET, respectively) on PDAC cells, promoting invasion toward and along nerves(92). Neuroactive ligand-receptor signaling also has implications for cancer-related pain, a common and severe symptom in PDAC patients. Pancreatic tumors modulate pain receptors (nociceptors) through the release of certain neurotransmitters and inflammatory molecules, exacerbating the experience of pain in patients. This is often due to the activation of the transient receptor potential vanilloid 1 (TRPV1) and other pain-related receptors expressed in pancreatic nerve fibers, which become hyperactivated as a result of neuro-ligand interactions. The persistent stimulation of these receptors amplifies pain signals to the brain. Neurotransmitter signaling also contributes to immune evasion. For instance, dopamine receptors on immune cells can suppress the immune response by inhibiting the activity of cytotoxic T-cells and promoting the recruitment of regulatory T-cells (Tregs), both of which support tumor immune escape (93).

The leukocyte transendothelial migration pathway refers to the movement of leukocytes (immune cells) across the endothelial barrier from the bloodstream into tissue, typically during immune surveillance or inflammation. However, in PDAC, this pathway is co-opted by the tumor to support its progression. PDAC is characterized by a highly immunosuppressive tumor microenvironment (TME), in part due to altered leukocyte migration. While immune cells, such as macrophages, neutrophils, and T-cells, migrate into the tumor, they are often reprogrammed by the tumor to support tumor growth rather than launch an anti-tumor immune response. Tumor-associated macrophages (TAMs), which migrate through this pathway, are frequently polarized into the M2 phenotype, which supports tumor growth, suppresses inflammation, and promotes tissue remodeling. The M2 macrophages secrete immunosuppressive cytokines like IL-10 and TGF-β, which inhibit effective anti-tumor immune responses. Regulatory T-cells (Tregs), which also migrate into the tumor via transendothelial migration, inhibit the activity of cytotoxic T-cells and natural killer (NK) cells, further reducing the immune system’s ability to attack the tumor. This leads to immune evasion, a hallmark of PDAC, where the tumor successfully suppresses immune surveillance mechanisms. The interaction between leukocytes and endothelial cells during migration is not only important for immune cell infiltration but also contributes to tumor angiogenesis and metastasis. Leukocytes, particularly TAMs, secrete factors like vascular endothelial growth factor (VEGF) and matrix metalloproteinases (MMPs), which promote angiogenesis and metastasis(94). Leukocytes migrating into the PDAC tumor microenvironment contribute to a chronic inflammatory state that paradoxically promotes tumor growth. While leukocyte infiltration typically signifies an immune response, in PDAC, the tumor co-opts this inflammatory response to sustain its own progression. The inflammatory cytokines secreted by leukocytes, such as TNF-α, IL-6, and IL-1β, promote cancer cell proliferation, survival, and resistance to apoptosis. Chronic inflammation within the PDAC microenvironment is associated with genetic instability, increased mutation rates, and the activation of oncogenic pathways, such as the NF-κB and STAT3 pathways, further accelerating tumor progression(95).

Both the neuroactive ligand-receptor interaction and leukocyte transendothelial migration pathways are intricately linked to the aggressive phenotype of PDAC. Their roles extend beyond simple signaling processes to become central to the immune suppression, perineural invasion, metastasis, and tumor-promoting inflammation that characterize PDAC(92). Neuroactive ligand-receptor interactions support tumor growth by activating oncogenic signaling, promoting nerve invasion, and contributing to cancer-related symptoms like pain, all of which are directly linked to the poor prognosis seen in PDAC patients (93). Leukocyte transendothelial migration, on the other hand, is key in shaping the immunosuppressive microenvironment, promoting angiogenesis, and aiding in metastasis, which are central to PDAC’s resistance to treatment and its high metastatic potential(94).

84.	Zeeh F, Witte D, Gädeken T, Rauch BH, Grage-Griebenow E, Leinung N, et al. Proteinase-activated receptor 2 promotes TGF-β-dependent cell motility in pancreatic cancer cells by sustaining expression of the TGF-β type I receptor ALK5. Oncotarget. 2016 Jul 5;7(27):41095–109.

85.	Siret C, Dobric A, Martirosyan A, Terciolo C, Germain S, Bonier R, et al. Cadherin-1 and cadherin-3 cooperation determines the aggressiveness of pancreatic ductal adenocarcinoma. Br J Cancer. 2018 Feb 20;118(4):546–57.

86.	Tsukahara M, Nagai H, Kamiakito T, Kawata H, Takayashiki N, Saito K, et al. Distinct expression patterns of claudin-1 and claudin-4 in intraductal papillary-mucinous tumors of the pancreas. Pathol Int. 2005 Feb;55(2):63–9.

87.	Ito T, Ito M, Shiozawa J, Naito S, Kanematsu T, Sekine I. Expression of the MMP-1 in human pancreatic carcinoma: relationship with prognostic factor. Mod Pathol. 1999 Jul;12(7):669–74.

88.	Rhim AD, Stanger BZ. Molecular biology of pancreatic ductal adenocarcinoma progression: aberrant activation of developmental pathways. Prog Mol Biol Transl Sci. 2010;97:41–78.

89.	Zhu W, Wang Y, Zhang D, Yu X, Leng X. MiR-7-5p functions as a tumor suppressor by targeting SOX18 in pancreatic ductal adenocarcinoma. Biochemical and Biophysical Research Communications [Internet]. 2018 Mar [cited 2024 Aug 12];497(4):963–70.

90.	Jiang, S.-H., et al. (2017). Serotonin enhances the proliferation of pancreatic cancer cells in vitro and in vivo. *Pancreas*, **46**(1), 12-19.

91.	Guo K, Zhao Y, Cao Y, Li Y, Yang M, Tian Y, Dai J, Song L, Ren S and Wang Z (2023), Exploring the key genetic association between chronic pancreatitis and pancreatic ductal adenocarcinoma through integrated bioinformatics. Front. Genet. 14:1115660

92.	Bapat, A. A., et al. (2011). Perineural invasion and neuropathic pain in pancreatic cancer. *Nature Reviews Cancer*, **11**, 695–707.

93.	Mantyh, P. W. (2004). Cancer pain and its impact on diagnosis, survival, and quality of life. *Nature Reviews Neuroscience*, **5**, 71-81.

94.	Beatty, G. L., & Gladney, W. L. (2015). Immune escape mechanisms as a guide for cancer immunotherapy. *Clinical Cancer Research*, **21**, 687-692.

95.	Noy, R., & Pollard, J. W. (2014). Tumor-associated macrophages: From mechanisms to therapy. *Immunity*, **41**, 49-61.
